# Development of an efficient micropropagation system for *Dioscorea bulbifera* L. and phytochemical profile of regenerated plants

**DOI:** 10.1186/s43141-022-00382-9

**Published:** 2022-07-15

**Authors:** Musadiq Hussain Bhat, Mufida Fayaz, Amit Kumar, Alamgir A. Dar, Ashok Kumar Jain

**Affiliations:** 1 School of Studies in Botany, Jiwaji University, Gwalior, 474011 India; 2 Institute of Ethnobiology, Jiwaji University, Gwalior, 474011 India; 3 Research Centre for Residue and Quality Analysis, Sher-e-Kashmir University of Agricultural Sciences and Technology (SKUAST-K), J, Srinagar, &K 180009 India

**Keywords:** Diosgenin, Conservation, Plant growth regulators, Nodal explant, HPTLC

## Abstract

**Background:**

The present study was designed to develop an improved in vitro regeneration system for *Dioscorea bulbifera* using mature nodal explants. Direct organogenesis from nodal segments was achieved by culturing the nodal explants on Murashige and Skoog medium supplemented with 3.5 mgl^−1^ 6-benzylaminopurine (BAP). Shoot multiplication was widely affected by the kind and concentration of plant growth regulators, and the sub-culturing of in vitro regenerated shootlets on fresh medium. After incubation for 4 weeks at optimum BAP concentration, cultures were transferred to secondary medium with BAP (optimized concentration) supplemented with different auxins.

**Results:**

On medium with 3.5 mgl^−1^ 6-benzylaminopurine, maximum regeneration (87 ± 1.85%) with an average shoot number of 2.0 ± 0.08, and length of 3.5 ± 0.04 cm were observed after 4 weeks of incubation. Maximum number of shoots (3.9 ± 0.21) was observed with 3.5 mgl^−1^ BAP in combination with 0.75 mgl^−1^ indole-3-acetic acid. The best root formation was observed on ½ MS medium supplemented with 0.75 mgl^−1^ α-naphthalene acetic acid, with 4.7 ± 0.03 roots per shoot. The well-rooted plantlets were successfully acclimatized in with 100% survival rate. The plantlets grew well with normal growth, flowering and showed, by High performance thin layer chromatography, almost same number of phytoconstituents compared with the mother plant.

**Conclusions:**

This is the first study on comparative phytochemical analysis of wild growing and in vitro regenerated plants of *D. bulbifera* which validates the medicinal and nutritional properties of in vitro raised plants.

## Background

*Dioscorea bulbifera* L. (Dioscoreaceae) is a glabrous non spiny annual climber, which grows up to 12 m or more. It is distributed globally throughout moist tropics and extending to warm temperature regions. It is mostly distributed in Asia and Africa in the wild and commonly naturalized in Central and South America, Nepal, and China. It is a source of diosbulbins as major phytochemicals [40, 44]. Other phytochemicals of immense importance include flavonoids, carotenoids, bafoudiosbulbins, steroidal saponins, and essential amino acids [6, 41]. Diosgenin (steroidal saponin) in this plant have gained interest in pharmaceutical industry as precursor for the synthesis of sex hormones, fertility control drugs, cardiatonic glucosides, and corticosteroids [3]. In India, it is used for the treatment of piles, syphilis, ulcers, dysentery, and inflammation [18, 42]. Various parts of this plant are used to treat various ailments such as tumors, leprosy, struma, sores, sore throat, and wounds worldwide [9, 23, 28]. The species exhibit anthelmintic, antioxidant, diuretic, rejuvenating, purgative, antidiabetic, and aphrodisiac properties [4]. *Dioscorea bulbifera* has a constricted germplasm base and is being continuously exploited for its nutritional and medicinal potential. Besides this, other anthropogenic activities and non-scientific harvesting also causes a severe threat to the survival of this species as it has low fruit setting rate and poor seed germination [1]. Vegetative propagation (through tuber and bulbils) is the common means of multiplication but insufficient to fulfil the demand of tribal people and pharmaceutical industry. Therefore, it is imperative to develop a rapid propagation method which can ensure increment of commercial production of *D. bulbifera* at commercial scales. Therefore, an attempt was made to develop an efficient and improved in vitro propagation protocol for *D. bulbifera* using nodal explants obtained from mature plant. In the present study, shoot regeneration rate was very high in comparison to the earlier studies on this plant. Although many reports on micropropagation of *D. bulbifera* are available, there is no report on comparative phytochemical analysis of wild growing and in vitro micropropagated plants of this plant in order to ensure the medicinal and nutritional properties of in vitro regenerated plants. The study described an efficient plant regeneration method by direct organogenesis of nodal segment explants, screening of secondary metabolites, and comparative analysis of mother plants and micropropagated plants.

## Methods

### Micropropagation

#### Explant source and surface sterilization of explants

Mature 1–2-cm-long nodal explants were collected from mature elite plants maintained in the Medicinal Plant garden, Jiwaji University, Gwalior. A voucher specimen (IOE-451) was deposited in the herbarium of Institute of Ethnobiology, Jiwaji University, Gwalior. The explants were washed for 15 min with running tap water. The explants were then washed with 4% (v/v) Tween-20 (SRL, Pvt. Ltd., Mumbai, India) for 5 min and dipped in 2% (w/v) Bavistin solution (BASF India Ltd., Mumbai, India) for 5 min and washed with autoclaved distilled water 3–5 times. Explants were then surface sterilized with freshly prepared 0.1% (w/v) aqueous HgCl_2_ (SRL, Mumbai, India) for 3 min with constant shaking in laminar flow cabinet. The explants were finally rinsed thrice with sterilized distilled water to remove HgCl_2_ traces prior to inoculation.

#### Medium composition, culture conditions, and shoot initiation

Murashige and Skoog [27] medium (MS) was fortified with BAP, KIN and TDZ ranging from 0.5 to 5.0 mgL^−1^, and 3% (w/v) sucrose (Himedia) as carbon source and 0.8% (w/v) agar (Himedia, India) as gelling agent. The media pH was maintained at 5.65 to 5.75 using 0.1 N NaOH or 0.1 N HCl. The medium (approx. 20–25 mL) was poured into 50 mL culture tubes or 100 mL culture flasks, before autoclaving at 121 °C and 15 psi pressure for 15–25 min The cultures were incubated at 25 ± 2 °C and 55 ± 2% relative humidity under 16/8 h (light/ dark) photoperiod in cool white fluorescent light (1800–2000 lux) intensity for shoot induction. After inoculation, these cultures were maintained for 4 weeks in incubation room. Regenerated multiple shoots were then transferred to the fresh medium after 4 weeks interval for further proliferation and elongation of developed shoots. The shoot multiplication was observed by counting of proliferated shoots. Further sub-culturing was made only on medium which showed maximum shoot multiplication.

#### Elongation and multiplication of shoots

In order to evaluate the effect of secondary medium on elongation and multiplication of shoots, the explants grown on medium supplemented with optimized concentrations of BAP and KIN were further transferred either to hormone-free MS medium or MS medium supplemented with BAP and KIN alone or in combination of indole-3-acetic acid (IAA), indole-3-butyric acid (IBA), or α-naphthalene acetic acid (NAA), ranging from 0.25 to 1 mgL^−1^ to evaluate the effect of the secondary medium on growing cultures for elongation and multiplication of shoots. The cultures were sub-cultured onto similar fresh medium every 4 weeks up to a maximum of four subcultures. These results were recorded after four subcultures.

#### In vitro rooting and acclimatization

For in vitro rhizogenesis, healthy elongated shoots having 2−3 nodes were cut out from the cultures and transferred to full or ½MS medium with 3% sucrose and 0.8% (w/v) agar fortified with different auxins (IAA, IBA, and NAA). Rooted plantlets removed from the culture medium were washed gently under running tap water to remove the agar traces. These plantlets were then transferred in plastic cups having sterilized mixture of soil, sand, and vermicompost (1:1:1) and sprinkled with ½MS basal solution (without inositol and sucrose) every 5 days for 2 weeks. These potted plantlets were covered with transparent plastic cups humidity maintenance and kept under the culture room conditions. After 30 days, the plantlets were placed in earthen pots containing normal garden soil and kept under shade in net house for advanced growth and development.

#### Experimental design and statistical analysis

Experiments were set up in a randomized block design (RBD) and each experiment had 10 replicates and repeated thrice. In each experiment, 10 explants were used for every treatment. Observations were recorded for the shoot induction, number of shoots, average shoot length, root induction, number of roots and average root length. The data was analysed by ANOVA and the significance of differences was computed by Duncan’s multiple range test (DMRT) at a 0.05% probability level by SPSS software (version 16.0).

### Phytochemical analysis

Various parts of *D. bulbifera* such as leaf, stem, and tuber were collected from mother plants maintained in the garden and in vitro regenerated plants and used for phytochemical analyses.

#### Quantitative phytochemical screening

Selected parts of *Dioscorea bulbifera* (leaf, stem, and tuber) were washed with tap water, shade dried at room temperature and powdered by an electrical blender. These samples were subjected to various analysis methods for quantification of various phytochemicals like total carbohydrate content, total sugar, free sugars and starch (Anthrone method), proteins [24], lipids [2], total phenols (Folin-Ciocalteau method), alkaloids (Harborne method), tannins (Folin Denis method), total flavonoid content (aluminium chloride colorimetric method), and saponins [31].

#### Thin layer chromatography

Aluminium plates (Merck 60F254) with pre-coated silica gel were used as a stationary phase. The solvent systems used as the mobile phase in TLC were hexane:ethyl acetate (7.2:2.9) and chloroform:acetic acid:methanol:water (6.4:3.2:1.2:0.8). Ten microliters of each extract was applied on TLC plates using capillary tube 2 cm above its bottom end. The plates were developed in different solvents separately. After developing, the plate was air dried and observed under UV at various wavelengths (254, 366, and 500 nm). Best solvent system was selected after spraying with anisaldehyde–H_2_SO_4_.

#### HPTLC fingerprinting of plant extracts

The best solvent system which showed good separation and maximum number of spots in TLC analysis was selected as the mobile phase for HPTLC. Analysis was performed on 10 cm × 10 cm pre-coated silica gel plates (60 F254, Merck, Germany). Prior to use, these plates were washed with methanol and dried at 120 °C for 5 min. One gram (each) of powdered samples was refluxed with 10 ml methanol and centrifuged at 3000 rpm for 5 min programmed by win CATS Planar Chromatography Manager for loading the samples. On each track 10 μl sample was applied. Six tracks were loaded on TLC plates with band length of 7 mm. The track spacing was 11.6 mm and scanning speed was 20 mm/s. Sample loaded plate was allowed to dry and then placed in TLC twin trough chamber for chromatogram development in the respective mobile phase up to 80–90 mm. Plate was removed from the developing chamber and total migration of solvent was marked immediately. This was followed by drying with dryer to remove the solvent traces. The images were captured at visible light, UV 254 nm and 366 nm in photo-documentation chamber (CAMAG Reprostar-3). The developed plate was sprayed with derivatizing agent anisaldehyde-sulphuric acid and dried at 100 °C in the hot air oven. The plate was photo-documented in UV 366 nm mode. Before derivatization, the plate was fixed in scanner stage (Camag TLC Scanner 3) and scanning was carried out at visible light, UV 254 nm and 366 nm (before and after derivatization). The peak table, peak display, and peak densitogram were noted. The data was evaluated using Win CATS Software.

## Results

### Micropropagation

#### Shoot regenration and establishment of cultures

**Fig. 1:**
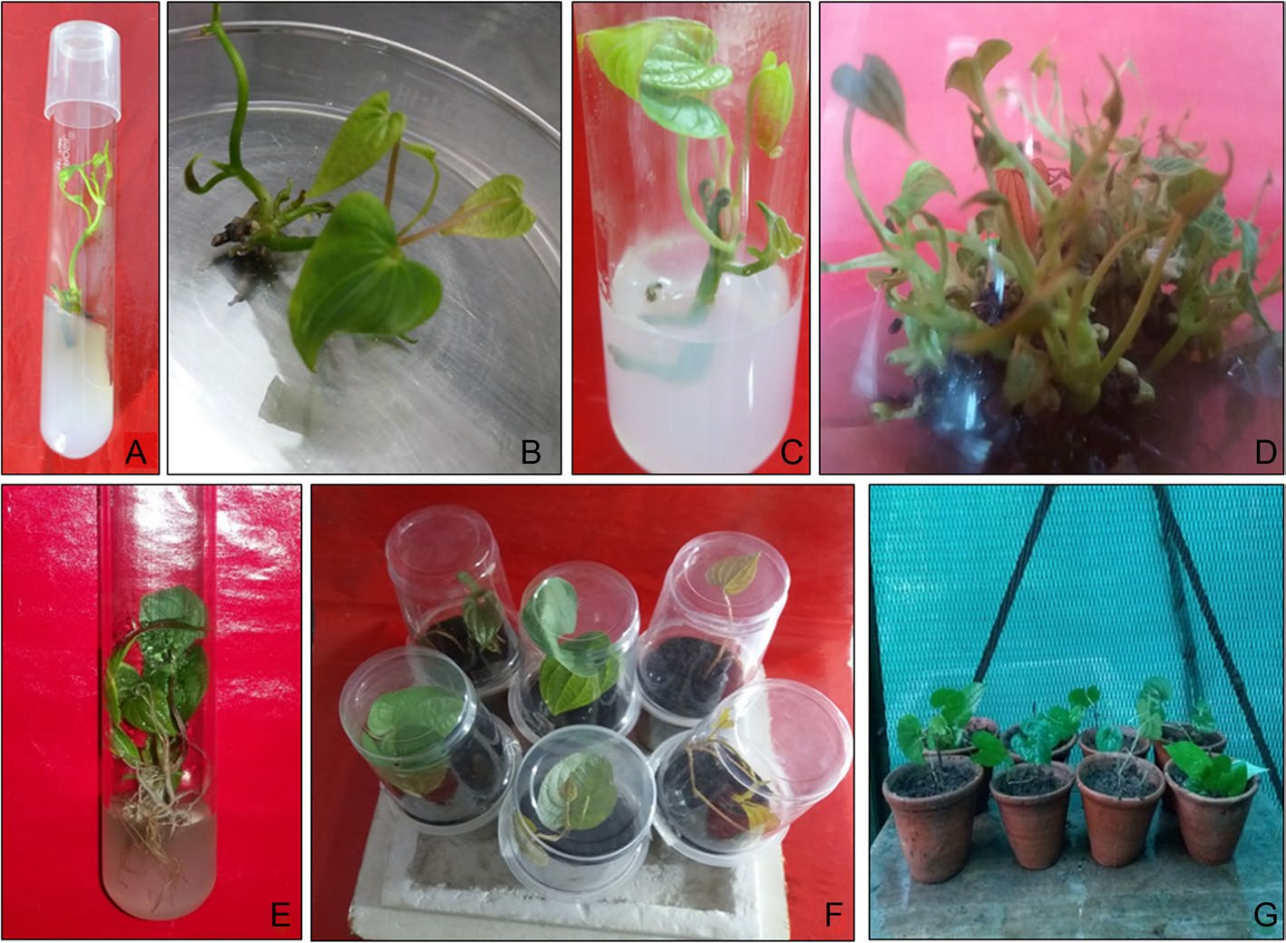
**A, B** Induction of shoots from the nodal explants on MS medium supplemented with 3.5 mg L^−1^ BAP. **C** Induction of shoots from the nodal explants on MS medium supplemented with 2.5 mg L^−1^ KIN. **D** Shoot multiplication on MS medium + 3.5 mg L^−1^ BAP and 0.75 mg L^−1^ IAA. **E** In vitro rooting on ½MS medium + 0.75 mg L^−1^ NAA. **F** Plantlets in plastic cups. **G** Acclimatized plantlets ready for field transfer

On MS medium supplemented with 3.5 mg L^−1^ BAP, maximum regeneration (87 ± 1.85%) with an average shoot number of 2.0 ± 0.08, and length of 3.5 ± 0.04 cm were observed after 4 weeks of incubation (Fig. 1A, B, Table 1). The rate of shoot induction was quite high in comparison to some earlier reports for *Dioscorea bulbifera*. The rate of obtained shoot induction was low when MS medium was augmented with KIN and TDZ with smaller shoot number and average shoot size.

**Table 1:** Effect of cytokinins on shoot induction from nodal segment explants

Concentration of cytokinins (mg L^**−1**^)			Frequency of regeneration (% ± SE)	Shoot number (mean ± SE)	Shoot length (cm) (mean ± SE)
BAP	KIN	TDZ			
00	–	–	0 ± 0.00	0.0 ± 0.00	0.0 ± 0.00
0.5	–	–	54 ± 1.85^fghi^	1.2 ± 0.08^efgh^	1.2 ± 0.13^egh^
1.0	–	–	54 ± 1.85^eghi^	1.4 ± 0.12^g^	1.1 ± 0.22^efg^
1.5	–	–	59 ± 1.85^fghi^	1.4 ± 0.06^g^	1.4 ± 0.32^egh^
2.0	–	–	52 ± 1.85^cefghij^	1.4 ± 0.18^g^	1.6 ± 0.07^egh^
2.5	–	–	63 ± 1.85^abdfghi^	1.6 ± 0.18^a^	2.8 ± 0.20^abcdehij^
3.0	–	–	72 ± 2.99^abcdegj^	1.7 ± 0.16^aij^	1.7 ± 0.11^be^
**3.5**	–	–	**87 ± 1.85**^**abcdefhij**^	**2. ± 0.08**^**abcdefhij**^	**3.5 ± 0.04**^**abcdefij**^
4.0	–	–	73 ± 3.33^abcdegj^	1.7 ± 0.13^aij^	3.5 ± 0.09^abcdefij^
4.5	–	–	76 ± 1.85^abcdeij^	1.3 ± 0.06^fgh^	1.4 ± 0.01^egh^
5.0	–	–	59 ± 1.84^dfghi^	1.2 ± 0.15^fgh^	1.5 ± 0.04^egh^
–	0.5	–	41 ± 1.85^defgh^	1.1 ± 0.08^ceg^	1.3 ± 0.68^bcdeg^
–	1.0	–	50 ± 3.21^deghj^	1.3 ± 0.06^e^	2.8 ± 0.38^ahij^
–	1.5	–	43 ± 1.85^degh^	1.6 ± 0.06^adfhij^	2.4 ± 0.17^aij^
–	2.0	–	67 ± 3.21^abcefghij^	1.2 ± 0.08^ce^	2.4 ± 0.14^aij^
–	2.5	–	87 ± 1.85^abcdfhij^	1.7 ± 0.11^abdfghij^	3.1 ± 0.16^afhij^
–	3.0	–	57 ± 4.90^acdeghij^	1.2 ± 0.11^ce^	1.8 ± 0.51^e^
–	3.5	–	85 ± 3.70^abcdfhij^	1.4 ± 0.05^ae^	2.6 ± 0.44^aij^
–	4.0	–	76 ± 1.85^abcdfgij^	1.3 ± 0.06^ce^	1.7 ± 0.08^be^
–	4.5	–	44 ± 3.21^defgh^	1.3 ± 0.14^ce^	1.2 ± 0.07^bcdeg^
–	5.0	–	39 ± 3.21^bdefgh^	1.2 ± 0.10^ce^	0.9 ± 0.06^bcdeg^
–	–	0.5	35 ± 1.85^c^	1.1 ± 0.08^bcdehi^	0.9 ± 0.06^cefghj^
–	–	1.0	35 ± 1.91^c^	1.3 ± 0.03^a^	0.5 ± 0.05^dehi^
–	–	1.5	46 ± 1.93^abdgij^	1.3 ± 0.05^a^	0.6 ± 0.05^acde^
–	–	2.0	37 ± 3.70^cj^	1.5 ± 0.03^afg^	0.9 ± 0.06^bcefghij^
–	–	2.5	39 ± 3.21^j^	1.4 ± 0.13^afg^	1.1 ± 0.05^abcdfghij^
–	–	3.0	41 ± 3.70^j^	1.1 ± 0.11^dehi^	0.6 ± 0.09^ade^
–	–	3.5	33 ± 0.00^c^	1.1 ± 0.11^dehi^	0.6 ± 0.01^ade^
–	–	4.0	41 ± 3.70^j^	1.4 ± 0.07^afg^	0.7 ± 0.03^abde^
–	–	4.5	33 ± 6.41^c^	1.4 ± 0.07^afg^	0.7 ± 0.01^bdej^
–	–	5.0	28 ± 3.21^cdefh^	1.3 ± 0.15	0.5 ± 0.01^adei^

Mean separation was analyzed by ANOVA using SPSS software (16.0) and significance of variations between the concentrations was studied using DMRT at 0.5 % level

#### Effect of plant growth regulators on shoot multiplication

Among the different formulations, BAP (3.5 mg L^−1^) in combination with 0.75 mg L^−1^ IAA improved the multiple shoot induction (87%) with maximum shoot number per explant (3.9) and shoot length (2.34 cm) after incubation of 3 weeks (Fig. 1D, Table 2). From the results, it is evident that BAP was highly effective for shoot regeneration as compared to KIN. The efficiency of auxins synergistically with cytokinins for multiple shoot regeneration followed the order of effectiveness IAA>IBA or NAA. All the three tested auxins showed varied multiple shoot induction at different concentrations (0.25–1.0 mg L^−1^). However, auxin concentration beyond 0.75 mg L^−1^ lead to reduced shoot induction and average shoot length and number.

**Fig. 2:**
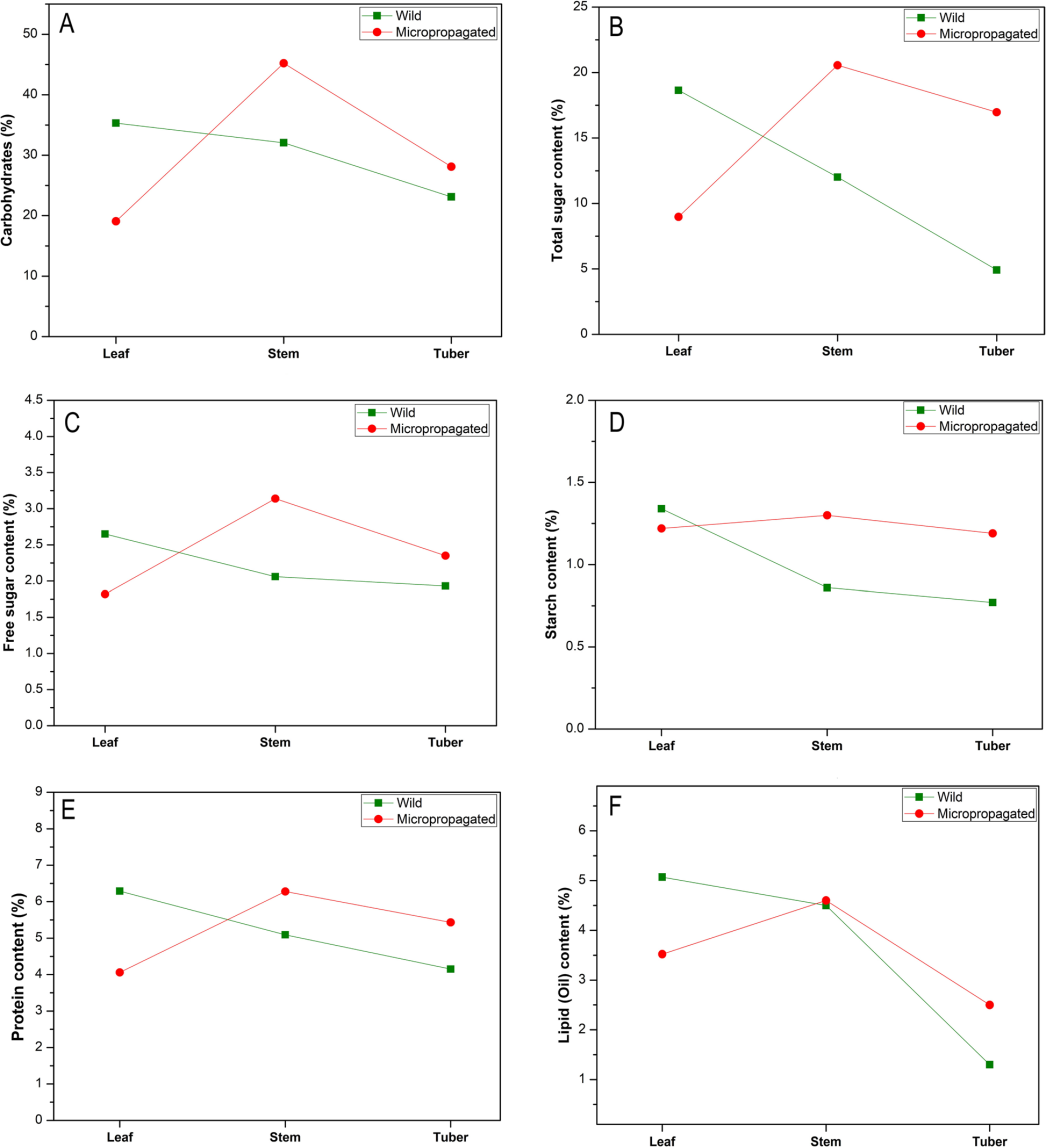
Quantification of primary metabolites in various parts of wild and micropropagated plants of *Dioscorea bulbifera*. **A** Carbohydrate content (%). **B** Total sugars (%). **C** Free sugars (%). **D** Starch (%). **E** Protein content (%). **F** Lipid content (%)

**Table 2:** Effect of selected BAP concentration and different auxin concentrations on shoot multiplication of *Dioscorea bulbifera* on MS medium

BAP (mg L^−1^)	IAA (mg L^−1^)	IBA (mg L^−1^)	NAA (mg L^−1^)	Frequency of shoot multiplication (% ± SE)	Shoot number (mean ± SE)	Shoot length (cm) (mean ± SE)
3.5	0.25	^−^	^−^	67 ± 3.33^cd^	2.0 ± 0.09^bc^	3.3 ± 0.10^cd^
3.5	0.50	^−^	^−^	70 ± 5.77^cd^	2.5 ± 0.12^acd^	3.5 ± 0.05^cd^
**3.5**	**0.75**	^−^	^−^	**87 ± 3.33**^**abd**^	**3.9 ± 0.21**^**abd**^	**4.8 ± 0.12**^**abd**^
3.5	1.0	^−^	^−^	43 ± 3.33^abc^	1.9 ± 0.08^bc^	2.4 ± 0.06^abc^
3.5	^−^	0.25	^−^	57 ± 3.33	2.0 ± 0.21^b^	2.1 ± 0.02^bd^
3.5	^−^	0.50	^−^	67 ± 3.33^cd^	2.7 ± 0.17^acd^	2.9 ± 0.05^acd^
3.5	^−^	0.75	^−^	53 ± 6.67^b^	2.3 ± 0.03^b^	2.1 ± 0.06^bd^
3.5	^−^	1.0	^−^	50 ± 5.77^b^	2.2 ± 0.10^b^	1.5 ± 0.03^abc^
3.5	^−^	^−^	0.25	73 ± 3.33^cd^	1.3 ± 0.13^bc^	3.2 ± 0.02^bcd^
3.5	^−^	^−^	0.50	80 ± 5.77^cd^	2.0 ± 0.15^a^	4.5 ± 0.09^acd^
3.5	^−^	^−^	0.75	50 ± 5.77^ab^	1.8 ± 0.32^a^	2.4 ± 0.11^ab^
3.5	^−^	^−^	1.0	43 ± 3.33^ab^	1.6 ± 0.19	2.2 ± 0.04^ab^

Mean separation was analyzed by ANOVA using SPSS software (16.0) and significance of variations between the concentrations was studied using DMRT at 0.5 % level

#### Effect of auxins on rooting

Among the different strengths of tested MS media, ½MS medium proved to be more effective. It was revealed that the maximum percentage of rooting (100%) with maximum number of roots (4.7) per shoot and average root length (4.1 cm) was observed on ½MS medium fortified with 0.75 mg l^−1^ NAA (Fig. 1E, Table 3). In this study, IAA or IBA was less effective than NAA in stimulating rooting.

**Table 3:** Effect of different auxins on in vitro root induction of *Dioscorea bulbifera* on ½MS medium

Auxins concentrations (mg L^−1^)		Frequency of rooting (% ± SE)	Root number (mean ± SE)	Root length (cm) (mean ± SE)
Control	0	0 ± 0.00	0.0 ± 0.00	0.0 ± 0.00
IAA	0.25	60 ± 5.77^b^	2.9 ± 0.28	1.3 ± 0.15^bc^
	0.50	93 ± 3.33^acd^	3.1 ± 0.19	2.8 ± 0.18^acd^
	0.75	67 ± 3.33^b^	3.0 ± 0.24	2.2 ± 0.03^abd^
	1.0	70 ± 0.00^b^	2.7 ± 0.10	1.3 ± 0.20^bc^
IBA	0.25	77 ± 3.33^cd^	2.3 ± 0.08^c^	1.6 ± 0.12^bcd^
	0.50	80 ± 0.00^cd^	2.5 ± 0.15^c^	2.1 ± 0.08^ac^
	0.75	97 ± 3.33^abd^	3.5 ± 0.05^abd^	4.1 ± 0.03^abd^
	1.0	67 ± 3.33^abc^	2.6 ± 0.14^c^	2.1 ± 0.02^ac^
NAA	0.25	70 ± 5.77^bc^	2.7 ± 0.23^c^	1.0 ± 0.05^bcd^
	0.50	90 ± 5.77^ad^	3.2 ± 0.11^c^	2.3 ± 0.03^ac^
	**0.75**	**100 ± 0.00**^**ad**^	**4.7 ± 0.03**^**abd**^	**4.1 ± 0.05**^**abd**^
	1.0	73 ± 3.33^bc^	2.8 ± 0.13^c^	2.1 ± 0.15^ac^

Values represent means ± SE. Means followed by the same letter within columns are not significantly different (*P* = 0.05) using Duncan’s multiple range test

#### Acclimatization

Out of rooted mature plantlets which were potted in plastic cups containing soil, sand, and vermicompost (1:1:1) placed in the green house, 100% survived after 30 days. The micropropagated plants showed identical morphology and stability, free from evident abnormalities (Fig. 1F, G).

### Phytochemical analysis

#### Quantitative estimation

**Fig. 3:**
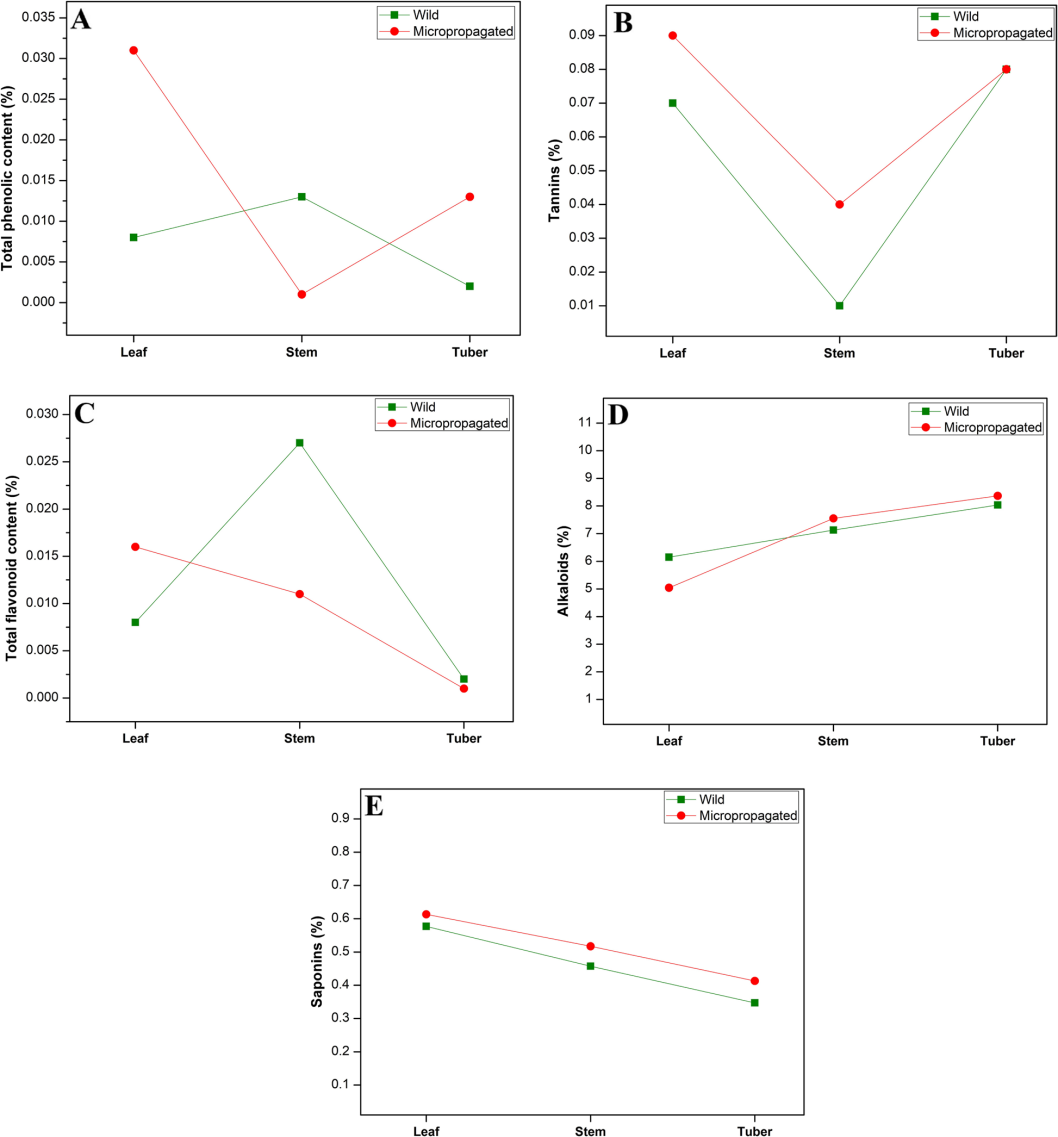
Quantification of secondary metabolites in various parts of wild and micropropagated plants of *Dioscorea bulbifera*. **A** Total phenolic content. **B** tannin content. **C** Total flavonoid content. **D** Alkaloids. **E** Saponins

The present study on quantitative estimation showed that alkaloids, proteins, phenolics, flavonoids, tannins, lipids and carbohydrates were found to exist abundantly in *D. bulbifera* extracts (Table 4). Mostly, the plant samples of regenerated plants accumulate higher amount of alkaloid, phenolic, flavonoid and tannin content wild growing plants. In the present study, although the stems of regenerated *D. bulbifera* plant showed increased quantities of phytochemicals like phenolics, tannins, and flavonoids, the leaves showed slightly smaller content of these phytochemicals than wild growing plants (Fig. 2). The results indicated that the leaf and tuber samples of regenerated plants possess higher phenolic content as compared to wild plants. In our study, lipid (oil) content was smaller in analysed parts of micropropagated plants than wild growing plants. All the analysed parts except leaves showed remarkable increment in the carbohydrate, total sugar, free sugar, starch and protein content in micropropagated plants than those of wild growing plants (Fig. 3).

**Table 4:** Quantitative estimation of phytochemicals from various parts of wild and in vitro raised *D. bulbibera* (%)

Phytochemical	Wild			Micropropagated		
	Leaf	Stem	Tuber	Leaf	Stem	Tuber
Carbohydrates	35.31 ± 0.22	32.06 ± 0.03	23.11 ± 0.08	19.07 ± 0.08	45.22 ± 0.06	28.10 ± 0.12
Total sugars	18.64 ± 0.32	12.01 ± 0.06	4.92 ± 0.02	8.98 ± 0.04	20.56 ± 0.32	16.97 ± 0.16
Free sugars	2.65 ± 0.03	2.06 ± 0.04	1.93 ± 0.02	1.82 ± 0.03	3.14 ± 0.03	2.35 ± 0.03
Starch	1.34 ± 0.03	0.86 ± 0.05	0.77 ± 0.01	1.22 ± 0.02	1.30 ± 0.02	1.19 ± 0.02
Protein	6.29 ± 0.03	5.09 ± 0.04	4.15 ± 0.04	4.06 ± 0.04	6.28 ± 0.01	5.43 ± 0.02
Lipid (Oil)	5.07 ± 0.04	4.50 ± 0.01	1.30 ± 0.02	3.52 ± 0.04	4.60 ± 0.03	2.50 ± 0.05
Phenolic compounds	0.008 ± 0.0002	0.013 ± 0.0001	0.002 ± 0.00	0.031 ± 0.0005	0.001 ± 0.00	0.013 ± 0.0001
Tannins	0.07 ± 0.001	0.01 ± 0.001	0.08 ± 0.002	0.09 ± 0.003	0.04 ± 0.002	0.08 ± 0.003
Flavonoids	0.008 ± 0.0002	0.027 ± 0.0004	0.002 ± 0.00	0.016 ± 0.0002	0.011 ± 0.0001	0.001 ± 0.00
Alkaloids	6.15 ± 0.03	7.13 ± 0.02	8.04 ± 0.04	5.04 ± 0.03	7.55 ± 0.04	8.37 ± 0.09
Saponins	0.58 ± 0.01	0.46 ± 0.01	0.35 ± 0.01	0.61 ± 0.02	0.52 ± 0.02	0.41 ± 0.01

Results are mean values ± standard error

**Fig. 4:**
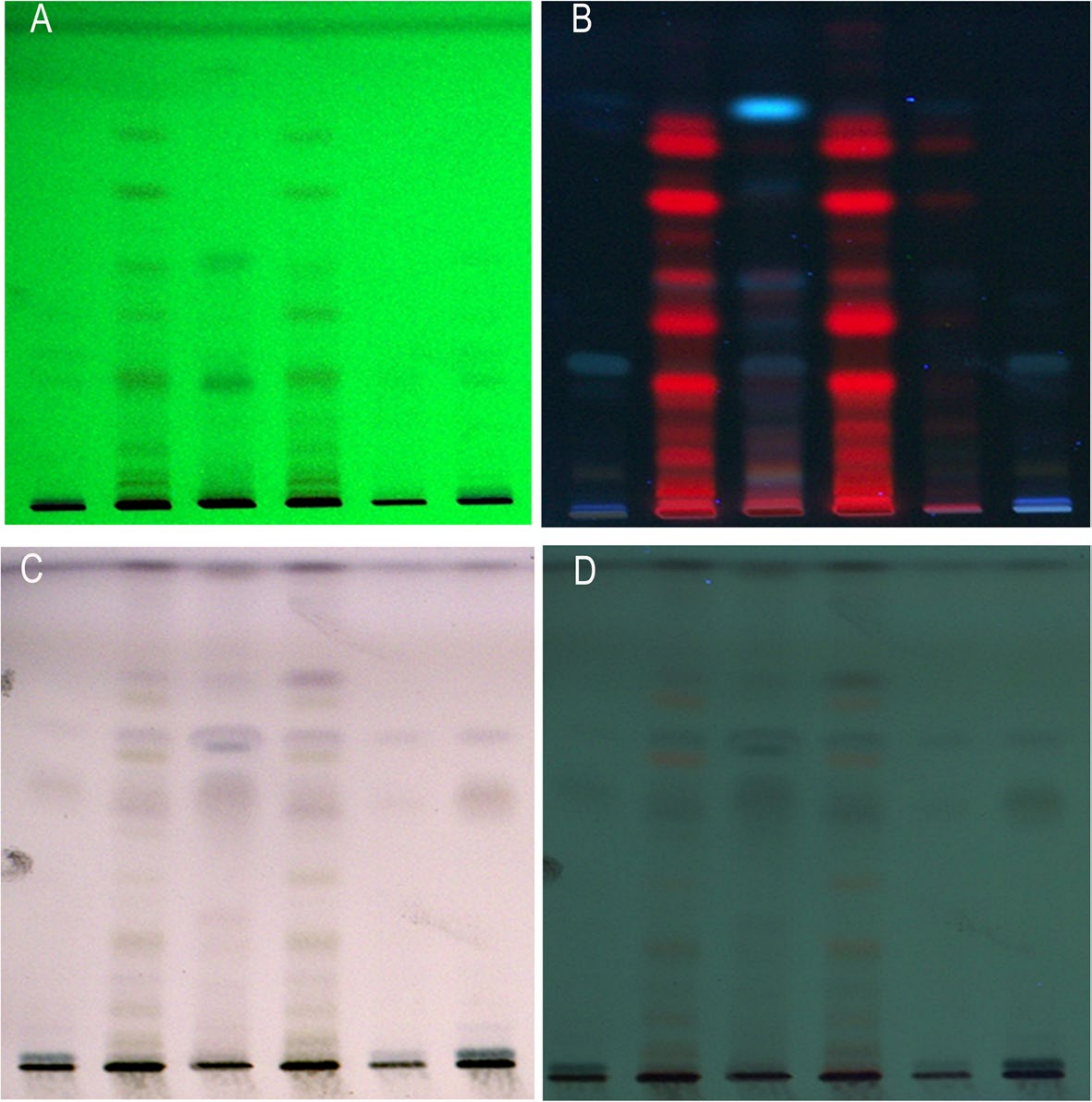
HPTLC chromotagram of methanolic extract of various samples (leaf, stem, and tuber) of *D. bulbifera*. **A** At 254 nm before derivatization. **B **At 366 nm before derivatization. **C** At 500 nm before derivatization. **D** At 366 nm after derivatization with anisaldehyde-sulphuric acid {solvant system:hexane:*ethyl acetate* (7.2:2.9)}. 1 = tuber (wild), 2 = leaf (wild), 3 = stem (wild), 4 = leaf (in vitro raised), 5 = stem (in vitro raised), 6 = tuber (in vitro raised)

#### Thin layer chromatography

TLC studies of the methanol extracts of *D. bulbifera* in solvent system 1 showed 12 spots for sample 1 [Tuber (wild)]; 14 spots for sample 2 [Leaf (wild)]; 10 spots for sample 3 [Stem (wild)]; 13 spots for leaves of in vitro raised plantlets; 10 spots for stem (in vitro) and 11 spots in tubers of in vitro raised plants. In solvent system 2, 7 spots were detected in tubers (wild); 10 spots in leaves (wild); 3 spots in stem (wild). Leaves, stem, and tubers of in vitro raised plants showed 7, 7, and 10 spots (Table 5). TLC plates showed different coloured phytoconstituents in methanol extracts of *D. bulbifera* in both the solvent systems. The results revealed presence of greenish, purple, pink, yellow, blue, and orange bands confirming the presence of alkaloids, flavonoids, tannins, glycosides, steroids, terpenoids, and saponins. The variation in Rf values gives an important evidence in recognizing their polarity. In this study, two different solvents were used, and better separation was achieved with hexane:ethyl acetate (7.2:2.9). TLC profile of methanol extracts showed remarkable results that gives the clue for the presence of various phytochemicals.

**Table 5:** Thin Layer chromatography of methanolic extract of various samples

Sample	No. of peaks and Rf values	
	Solvent system 1 Hexane:ethyl acetate (7.2:2.9)	Solvent system 2 Chloroform:acetic acid:methanol:water (6.4:3.2:1.2:0.8)
1	**(12)** 0.06, 0.10, 0.18, 0.27, 0.41, 0.49, 0.57, 0.60, 0.70, 0.80, 0.89, 1.15	**(7)** 0.16, 0.23, 0.25, 0.33, 0.44, 0.78, 0.82
2	**(14)** 0.09, 0.19, 0.24, 0.37, 0.43, 0.51, 0.60, 0.75, 0.79, 0.87, 0.01, 1.12, 1.16, 1.18	**(10)** 0.9, 0.13, 0.20, 0.24, 0.31, 0.42, 0.52, 0.65, 0.85, 0.91
3	**(10)** 0.07, 0.17, 0.25, 0.34, 0.42, 0.75, 0.82, 0.87, 1.01, 1.12	**(3)** 0.43, 0.77, 0.88
4	**(13)** 0.04, 0.11, 0.18, 0.25, 0.39, 0.43, 0.51, 0.53, 0.75, 0.87, 1.02, 1.08, 1.19	**(7)** 0.12, 0.16, 0.26, 0.32, 0.43, 0.85, 0.91
5	**(10)** 0.11, 0.26, 0.35, 0.39, 0.46, 0.58, 0.73, 0.87, 0.98, 1.12	**(7)** 0.11, 0.17, 0.27, 0.34, 0.43, 0.78, 0.89
6	**(11)** 0.07, 0.12, 0.18, 0.23, 0.26, 0.33, 0.37, 0.39, 0.76, 0.87, 1.14	**(10)** 0.60, 0.15, 0.27, 0.34, 0.43, 0.53, 0.69, 0.84, 0.90, 1.05

*1* = tuber (wild), *2* = leaf (wild), *3* = stem (wild), *4* = leaf (in vitro raised), *5* = stem (in vitro raised), *6* = tuber (in vitro raised)

#### HPTLC fingerprinting

HPTLC fingerprinting profile indicated that these contain appreciable number of active phytoconstituents in their methanolic extracts by showing various color bands at various wavelengths with specific solvent systems (mobile phases), representing the presence of particular phytocompounds. The HPTLC chromatograms indicate that all constituents in the analysed samples were clearly separated without any tailing and diffuseness. HPTLC fingerprinting profile of various parts (leaves, stem, and tubers) of both wild growing and in vitro raised *D. bulbifera* plants at various wave lengths showed good similarity in their components. A comparative screening of methanolic extracts of samples of both wild and micropropagated plants exhibited multiple bands with different Rf values; where at 254 nm (Fig. 4A) 7 bands were observed in extract of its tuber (wild) and 8 bands in tuber (in vitro raised). Leaf samples (wild) showed 11 bands, while as 10 bands were observed in leaves of micropropagated plants. Extracts from stem (wild) displayed 7 bands, contrastingly when compared to that of stem of micropropagated plant, only 4 peaks were observed. At 366 nm, methanol extract of tuber (wild) showed 3 bands, while as in vitro raised tubers showed 2 bands. Likewise, stem extracts (wild) exhibited 7 bands and only 5 bands were recorded in stem extract (micropropagated) (Fig. 4B). At 500 nm, same number of bands (7) was displayed by both the tuber samples (wild and micropropagated). Methanolic extracts of both leaf samples displayed 12 bands each. However, stem extracts (wild) displayed more bands (9) than that of stem of micropropagated plants (6) (Fig. 4C). However, at 366 nm after derivatization, the extracts of *D. bulbifera* tubers obtained from wild and micropropagated plants displayed 12 and 11 bands. Similarly, at 366 nm after derivatization, the leaf (wild) extracts revealed 14 bands and that of micropropagated plants showed 13 bands. On the other hand, stem (wild and micropropagated) showed 3 bands each. In comparison to the banding pattern at 245 and 366 nm, screening of all the extracts at 500 nm showed maximum number of bands. Similarly, while screening of extracts at 366 nm (after derivatization), the maximum bands were recorded in comparison to the banding patterns observed at 254, 366, and 500 nm (Fig. 4D). These results indicate the presence of similar nature of phytoconstituents in both types of samples.

## Discussion

*Dioscorea bulbifera* is a seasonal medicinal plant, which appears with the beginning of rainy season. Explants collected in August–September responded well in vitro. Nodal segments are the preferred explant type used in numerous investigations for culture initiation of medicinal plants [14, 22, 29, 34, 38]. These explants are found to be superior for in vitro regeneration with retained genetic stability [37]. The rate of shoot induction is quite high on MS medium supplemented with BAP in comparison to medium augmented with KIN and TDZ. BAP has been reported as the most effective cytokinin in a large number of in vitro studies. Structural stability and easy assimilation of BAP by the plant cells make it prominent among the cytokinins. Similar results have also been reported in many other plant species like *Eclipta alba* [33], *Stevia rebaudiana* [43], *Ceropegia evansii* [8] *Bacopa monnieri* [19] and *Morinda citrifolia* [38] in which explants from the medium containing BAP exhibited greater potential for shoot regeneration than those from the media supplemented with other cytokinins (KIN and TDZ). Relative concentration of cytokinins and auxins in the growth medium also regulates the shoot induction and differentiation. The efficiency of auxins to promote shoot differentiation when combined with cytokinins was also found conspicuous in this study. A high concentration of cytokinin when fortified with low auxin concentrations showed promising results for the shoot induction and multiplication in *D. bulbifera.* From the results, it is evident that BAP was highly effective for shoot regeneration as compared to KIN, which is in agreement with the study of Lupi and his coworkers on *Helianthus annus* [25]. Synergistic effect of BAP when combined with an auxin has been confirmed in many medicinal plant species from the genus *Dioscorea*, viz. *Dioscorea alata*, *D. hispida*, and *D. fordii* [5, 12, 45]. In compliance with these findings, the present study also demonstrated enhanced shoot induction obtained using a combination of a cytokinin and auxin. The efficiency of auxins synergistically with cytokinins for multiple shoot regeneration followed the order of effectiveness IAA>IBA or NAA. The advantage of IAA in comparison to other auxins (IBA and NAA) for shoot multiplication has also been reported in *Ceropegia evansii* [8] and *Nilgirianthus ciliates* [32]. All the three tested auxins showed varied multiple shoot induction at different concentrations. However, auxin concentration beyond 0.75 mg l^−1^ lead to reduced shoot induction and average shoot length and number. These results are quite similar with the recent studies in *Ceropegia evansii* [8], in which the stimulatory effect of BAP and IAA combination resulted in the increased regeneration frequency and multiple shoot formation. Among the different strengths of MS media tested, ½ MS medium proved to be more effective. The favorable effects of low concentrations of MS media along with auxins have been observed to induce rooting in shoots of *Myrica esculenta* [7] and *Rauwolfia serpentina* [35]. In this study, IAA or IBA were less effective than NAA in stimulating rooting. Similar results were also found in previous studies on some medicinally important plant species such as *Abutilon ranadei* [39] and *Nopalxochia ackermannii* [13].

Quantitative estimation showed that the in vitro plant samples accumulate higher amount of alkaloid, phenolic, flavonoid, and tannin content in comparison to samples of wild growing plants. Few workers have reported the higher quantities of phytochemicals mostly secondary metabolites in the micropropagated plants, such as in *Tulbaghia violacea* and *Thymus lotocephalus* [10, 30]. In the present study, although the in vitro regenerated plant samples showed increased quantities of phytochemicals, phenolic, tannin, and flavonoid content in stem and leaves (tannin content) were found in slightly smaller amounts. The results indicated that the leaf and tuber samples of in vitro regenerated plants possess higher phenolic content in comparison to wild plant. Similar results have been observed in some plant species like, *Saussurea involucrate* [17] and *Habenaria edgeworthii* [16] where in in vitro plants, phenolic content was higher than those of wild plant. In our study, lipid (oil) content was decreased in analyzed parts of micropropagated plants than wild growing plants. However, Melendez et al. [26] showed the improved essential oil contents of in the micropropagated plants than the in vivo plants of *Turnera diffusa*.

TLC plates showed different coloured phytoconstituents in methanol extracts of *D. bulbifera* in both solvent systems. The results revealed presence of greenish, purple, pink, yellow, blue, and orange bands confirming the presence of alkaloids, flavonoids, tannins, glycosides, steroids, terpenoids, and saponins. The variation in Rf values gives an important evidence in recodnizing their polarity. It also helps out to select the appropriate solvent for separating compounds by chromatographic technique. Solvent mixture of with varying polarity in different ratios can be utilized for separating pure compounds in plant extracts. Selection of a proper solvent mixture for a specific plant extract can only be attained by investigating the Rf values of compounds in diverse solvent systems. TLC profile of methanol extracts showed remarkable results that gives the clue for the presence of various phytochemicals. In the literature data, it has been reported that solvent system hexane:ethyl acetate (different ratios) has been successfully utilized for the better separation of phytochemicals in various plant species like *Euphorbia pulcherrima* [36] and *Alpinia galangal* [46].

A simple HPTLC method has been standardized for obtaining the fingerprint profile of various parts of *D. bulbifera* from two different sources (wild and regenerated). HPTLC fingerprinting of methanolic extracts of various parts (leaf, stem and tuber) of wild growing and in vitro regenerated *D. bulbifera* plants was first time established. HPTLC fingerprinting profile indicated that these contain appreciable number of active phytoconstituents in their methanolic extracts by showing various color bands at various wavelengths with specific solvent systems (mobile phases), representing the presence of particular phytocompounds. The HPTLC chromatograms indicate that all constituents in the analyzed samples were clearly separated without any tailing and diffuseness. HPTLC fingerprinting profile of various parts of both wild growing and in vitro raised *D. bulbifera* plants at various wave lengths showed good similarity in their components. In future, these findings could be helpful for herbal drug standardization. The mobile phase used for HPTLC was relatively high polar to separate the bioactive compounds*.* Many earlier reports have also suggested mobile phase having high polarity solvents for successful separation of the compounds in many plant species [15, 20]. Similar studies have been carried out by many workers on various plant species like *Azadirachta indica* [11] and *Tragia plukenetii* [21]. Further work is required to depict the individual phytochemical constituents and their quantitative evaluation using marker compounds to fix standards for this plant species.

## Conclusions

Micropropagation technique can be used as a substitute system not only to produce the plantlets on large scale in a short period but also to increase the production of the primary and secondary metabolites in medicinal plants. Present findings have evidently suggested the synergetic and positive effects of BAP and IAA on *D. bulbifera* cultures enhanced shoot induction and proliferation. Plant growth regulators and in vitro conditions optimized for shooting and rooting in *D. bulbifera*, led to the establishment of an improved, efficient and economical protocol for the micropropagation of this plant. All in vitro produced shoots were rooted successfully by rhizogenesis, which could reduce the time, and cost of plantlet production. It could be a productive method for the large scale production of this seasonal, vulnerable, and highly important plant species. Moreover, the developed protocol could also be used to increase production of bioactive metabolites as revealed by phytochemical study. It is concluded from the present findings that the study may aid to mark, identify, and standardize active compounds for the production of new drug formulations for further research.

## Data Availability

All data generated or analyzed during this study are included in this published article [and its supplementary information files].

## Abbreviations

BAP: 6-Benzyl amino purine
HPTLC: High performance thin layer chromatography
IAA: Indole 3-acetic acid
IBA: Indole 3-butyric acid
KIN: Kinetin
Lux: Luxuriant
M: Molarity
MeOH: Methanol
min: Minutes
MS: Murashige and Skoog
N: Normality
Na2EDTA: Ethylene diamine tetra acetic acid
RT: Retention time
TDZ: Thidiazuron
TLC: Thin layer chromatography
